# Appraisal of Chicken Production with Associated Biosecurity Practices in Commercial Poultry Farms Located in Jos, Nigeria

**DOI:** 10.1155/2016/1914692

**Published:** 2016-04-21

**Authors:** C. V. Maduka, I. O. Igbokwe, N. N. Atsanda

**Affiliations:** Faculty of Veterinary Medicine, University of Maiduguri, Maiduguri 600001, Nigeria

## Abstract

A questionnaire-based study of chicken production system with on-farm biosecurity practices was carried out in commercial poultry farms located in Jos, Nigeria. Commercial and semicommercial farms had 75.3% and 24.5% of 95,393 birds on 80 farms, respectively. Farms using deep litter and battery cage systems were 69 (86.3%) and 10 (12.5%), respectively. In our biosecurity scoring system, a correct practice of each indicator of an event scored 1.00 and biosecurity score (BS) of each farm was the average of the scores of biosecurity indicators for the farm, giving BS of zero and 1.00 as absence of biosecurity and optimal biosecurity, respectively. Semicommercial farms had higher BS than commercial farms. The flock size did not significantly (*p* > 0.05) affect the mean BS. Disease outbreaks correlated (*r* = −0.97) with BS, showing a tendency of reduction of disease outbreaks with increasing BS. Outbreaks were significantly (*p* < 0.05) associated with deep litter system. In conclusion, the chicken production system requires increased drive for excellent biosecurity practices and weak points in the biosecurity could be ameliorated by extension of information to farmers in order to support expansion of chicken production with robust biosecurity measures that drastically reduce risk of disease outbreak.

## 1. Introduction

Entrepreneurial initiatives in the commercial poultry (chicken) industry increased in recent time in Jos, Nigeria, in response to national animal protein demand and as a result of the mild climatic environment and apparent profitability of the business. The industry is characterized by low to medium integration of inputs representing the downstream poultry sector where the flock stocking is low and involves raising day-old chicks, supplied by integrated farms, to mature weight (broilers) or egg-laying (laying hens) using commercial feeds and veterinary products from retail outlets [1, 2]. The chickens are fully housed in intensive management system and often reared on deep litter within urban and periurban locations. The production system is oriented towards profitability and the efforts to reduce cost of production may impact negatively on the system through neglect of important pivotal elements of poultry preventive health. Communicable diseases and problems related to feeding constitute the major constraint to profitable chicken production in the locality [2]. Therefore, disease prevention and control are veritable components of commercial poultry production. Biosecurity refers to principles engaged in reducing the chance of introduction and spread of pathogens within and between farms by preventing infectious agents from entering (bioexclusion) or exiting (biocontainment) the farm and the principal elements are segregation, traffic control, cleaning, and disinfection [3, 4]. An effective biosecurity has conceptual, structural, and operational frameworks which involve housing design and construction with management procedures that keep the flock free from infectious diseases [5–7]. The application of biosecurity is affected by small-holder, farm, social, and community characteristics [7, 8]. There have been reports of breaches in biosecurity measures in poultry production systems in parts of Nigeria because of lack of awareness and failure to implement components of biosecurity [9–11] resulting in frequent outbreaks of diseases which drastically reduce profit or lead to capital loss in the industry [2, 12]. The operational cost of biosecurity is usually low and there is a high benefit-cost ratio [4, 7, 13], but inadequate implementation of biosecurity measures may be due to insufficient motivation and lack of understanding of its economic benefits [14].

We hypothesized that the structure of chicken production in poultry farms, in the study area, was commercially oriented, family-based, and income-generating investment, which were set up as small-holder, farm-based intensive production system with some deficiencies in biosecurity practices and constraints associated with disease outbreaks [2]. The objective of this study, therefore, was to appraise chicken production along with the on-farm biosecurity practices (consistent with standard bioexclusion and biocontainment protocols) in commercial poultry farms located in Jos, Nigeria, and to assess whether some characteristics of the farms affect biosecurity rating and effectiveness in reducing outbreaks of communicable disease.

## 2. Materials and Methods

### 2.1. Study Area and Poultry Farms

**Figure 1:**
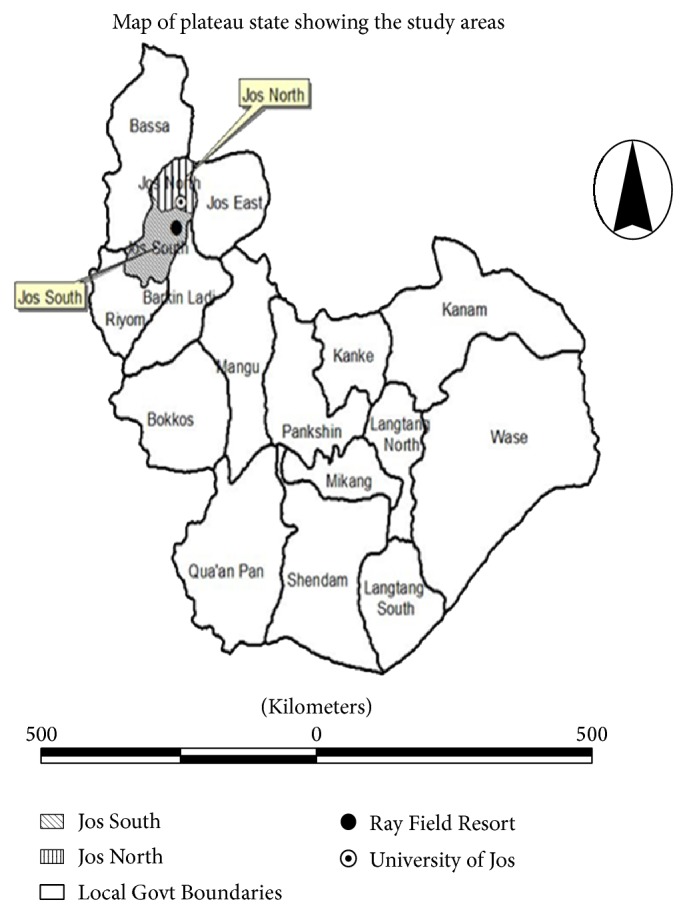
Map of Jos showing Jos North and South Local Government Areas among other Local Government Areas in Plateau State, Nigeria (Source: http://news.bbc.co.uk/2/hi/africa/8468456.stm. Accessed 11 September 2014).

The study was conducted in Jos North and South Local Government Areas located between 9°56′N and 8°53′E in Plateau State, Nigeria (Figure 1). The locations of 80 poultry farms were identified in the area through veterinarian-clientele relationship.

### 2.2. Questionnaire Construction

A questionnaire was used to gather information from 80 poultry farms on the following:

- Poultry farm production system.
- Biosecurity events outside the premises, at the farm boundary, between the farm boundary and poultry house, and within the poultry house.
- Outbreak of any communicable disease causing mortality after application of biosecurity in the current stocking cycle. The disease was syndromally diagnosed by the consulting veterinarian and included Marek disease, coccidiosis, infectious bursal disease, avian influenza, Newcastle disease, infectious coryza, fowl pox, fowl cholera, or egg drop syndrome after postmortem examination.

The questionnaire was structured to obtain “yes” or “no” answers and only open for specific additional responses for clarity of answers.

### 2.3. Filling the Questionnaire

The questionnaire was filled by the farmer or farm personnel during farm visits conducted in February–July 2014 and validated by information obtained after observation of the farm environment, interview of the farmer, and checking of farm records on the farm and in the veterinary clinic where the farm was registered. The validation was necessary in order to eliminate response bias which could weaken the strength of questionnaire survey [15].

### 2.4. Biosecurity Scoring System

A biosecurity scoring system was developed from the indicators of biosecurity events observed in the evaluation of biosecurity practices on the farm as previously reported [5, 8, 16–23]. The biosecurity indicators were 36 and the correct practice of each indicator was scored as 1.00. The biosecurity score (BS) of each farm was the average of the scores of the biosecurity indicators for the farm, giving a BS of zero and 1.00 as absence of biosecurity and optimal biosecurity, respectively.

### 2.5. Statistical Analysis

Data were collected from the questionnaire to obtain the number of positive responses for every item on the questionnaire as well as the number and ages of birds involved. The number of positive responses from respondents on each item was also presented as a percentage of total number of respondents and the response rate for the item was calculated as a percentage of the number of farms administered with questionnaires. The BS of farms were summarised as means and standard deviations. The associations and relationships were assessed using Chi-square and Pearson's correlation, respectively; and variations in means were assessed by one-way ANOVA with Tukey post hoc test using computer software (GraphPadInstat: http://www.graphpad.com/apps/index.cfm).

## 3. Results

### 3.1. Chicken Production System

The number of farms and chickens involved with associated reasons for poultry production, type of poultry, flock size, age of birds, housing system, sources of day-old chicks (DOC), and feed are presented in Tables 1(a) and 1(b). Very few farms (3.7%) produced chickens for family use only (consumption), but many farms (37.5%) produced for family use and marketed the surplus. The highest proportion of farms (58.8%) produced for strictly commercial purposes. Farms made incomes from sale of live chickens and table eggs to wholesale buyers, manure (faecal droppings) to crop farmers, and dead birds to dog breeders and keepers. The number of birds on strict commercial farms was 75.3% of the total number (95,393) of birds on 80 farms; whereas 24.5% of the birds were on semicommercial farms. Fifty-one (63.5%) farms raised only layers while 17 (21.2%) farms had both broilers and layers. Cockerel-layer and broiler farms were 1 (1.2%) and 11 (13.8%), respectively. The chicken population consisted of layers (48.8%), broilers (3.9%), broilers and layers (46.8%), or cockerels and layers (0.5%). Farms with flock size of 200–500 birds (36.2%) were most common followed by those with 501–1000 (25.0%) and 1001–2000 (18.8%) birds. The flock sizes with 30.7%, 25.3%, 16.5%, and 15.7% of the total bird population (TBP) were >10,000, 1,001–2,000, 501–1,000, and 2,000–10,000 birds per farm, respectively. Most birds (69.7%) were ≥18 weeks old in 54 (67.5%) farms while those that were <13 weeks old (28.3%) were in 23 (28.8%) farms. The older birds were usually layers and the younger ones were either broilers or growing layers. The farms using deep litter system were 69 (86.3%) housing 77.1% of TBP. Battery cages were used solely in 10 (12.5%) farms or in combination with deep litter in 1 (1.2%) farm with 7.9% and 15.0% of TBP, respectively. The day-old chicks, reared in 28 (35.0%) and 16 (20.0%) farms having 39.1% and 32.1% of TBP, were sourced from Zartech and Obasanjo farms, respectively. Few farms (*n* = 2–7, 2.5–8.7%) had other sources of DOC consisting of Agrited, ECWA, Amo, and Chi farms. Feeds used were commercial type in 68 (85%) farms housing 86.9% of TBP. The frequently used brands were Livestock, Vital, and Hybrid feeds in 19 (29.4%), 18 (26.5%), and 16 (23.5%) farms housing 41.4%, 14.9%, and 12.8% of TBP.

### 3.2. Biosecurity Appraisal

The frequencies of positive responses on indicators of biosecurity events in chicken-producing poultry farms with validated responses on a structured questionnaire are in Table 2. The biosecurity indicators that had >90% positive responses included presence of good storage facility, appropriate carcass disposal, rodent-proof facility, separation of poultry types and birds of varying ages, proper ventilation and availability of clean water, appropriate and dry bedding, washing and disinfection of poultry houses prior to restocking, regular washing of feeders and drinkers, and isolation of sick birds. The positive responses dropped to 80–90% in terms of awareness of the scope of appropriate biosecurity practices, fencing with gate, use of only certified commercial feed, washing hand and showering before and after handling birds, frequent changing of bedding, and practice of all-in all-out management. The positive responses further dropped to 60–80% regarding stocking DOC from certified sources, regularly disinfecting feeders and drinkers, use of functional footbath at the entrance of the poultry house, chemoprophylactic treatments of apparently healthy birds, and consulting veterinarians in the event of health challenges on the farm. Positive responses were 50–60% in the aspects of providing parking lot outside the farm premises and functional footbath at farm boundaries. Positive responses decreased to <50% in appropriate biosecurity practices of washing and disinfecting vehicles that drive into farm premises and residence of farm workers within the premises. Some farmers (47.4%) allowed visitors into the poultry premises. Positive responses for inappropriate biosecurity practices were <32% in situations involving clustering of farms (26.6%), water bodies for migratory birds in the neighbourhood (20.2%), acquisition of second-hand equipment (6.3%), on-farm carnivorous pets (8.9%), necropsies (31.2%), and allowing of birds to occasionally move out of their pens (3.8%).

Biosecurity scoring of 63 farms, with ≥34 validated responses related to biosecurity, showed that the farms had a mean BS of 0.80 ± 0.10 with minimum and maximum scores of 0.50 and 0.94, respectively.  Table 3 presents the effects of reason for keeping poultry and flock size on biosecurity scores. Mean BS was significantly (*p* < 0.05) affected by reason for poultry production, with semicommercial farms having higher BS than strictly commercial farms and noncommercial farms having comparable mean BS (*p* > 0.05) with strictly commercial farms. The flock size did not influence the mean BS (*p* > 0.05), but the highest mean BS was in farms with >10,000 birds per farm.  Table 4 presents frequencies of farms with various classes of BS in relation to occurrence of disease outbreak. The farms classified with good (0.50–0.70), very good (0.71–0.90), and excellent (0.91–1.00) BS consisted of 11 (17.5%), 45 (71.4%), and 7 (11.1%) farms, respectively. Disease outbreaks negatively correlated (*r* = −0.97) with BS showing that there was a tendency of reduction of disease outbreaks with increasing BS. Outbreaks occurred in 38 (55.1%) out of 69 farms using deep litter and no outbreak occurred in 10 farms using battery cages (see Table 5). There was significant (*p* < 0.05) association of outbreak with deep litter system.

## 4. Discussion

This study has provided the first expanded data base on the structure of chicken production system in Jos, Nigeria. Although a strictly commercial farm-based approach is the mainstay of the production system, a large contribution to the production capacity was made from family production which was usually semicommercial. Earlier reports in Nigeria indicated the preponderance of small-scale family chicken production as a means of reducing food insecurity and improving family income [2, 9, 18, 24–27]. The flock size per farm was frequently <1000 with the commonest flock size of 200–500, but aggregate population of chickens in this type of holding was just 38.3% of TBP. Previous reports, in the locality, indicated the most frequent flock size to be <200 [2] and <100 [26]. Family farms in other parts of Nigeria were reported to mostly have flock sizes of <200 [9] and ≤500 [27]. The production capacity increased when the flock sizes were >1000 such that the contribution to poultry sector was greater with larger than smaller farms. Thus, efficiency of production was expected to increase in larger farms as regards measures to mitigate production losses.

In our study, there were more layer than broiler farms and more layers than broilers in the TBP. Muhammad et al. [26] reported more broiler than layer farms, but more layers than broilers in stocking capacity. The larger population of birds, at the age of egg-laying compared to other ages, indicated that layers were more numerous in the bird population. Most of the birds were housed on deep litter as similarly reported by others [2, 9, 26]. More than 70% of DOC were obtained from two hatcheries and used by 55% of the farms indicating that the DOC suppliers probably had larger supply capacity outlet in Jos. The feeds were sourced from commercial feed producers by 85% of farms with 86.9% of TBP and three feed firms had the largest market perhaps because of logistic advantages. Therefore, farmers prefer commercial feeds to on-farm feed preparation because of, probably, better feed efficiency and cost effectiveness with commercial than on-farm feeds. Other DOC and feed suppliers are vital to the supply chain and may be gaining more market space as the production system expands.

The level of awareness of biosecurity practices among poultry farmers in Jos was high and as a result, no farm had poor BS of <0.5. Rather, some farms had good or excellent BS and most of them had very good BS. The awareness of the need for biosecurity is often elicited by veterinary advice which comes along with veterinary services during disease outbreaks [28]. Inadequate understanding of the scope of biosecurity practice remains the hindrance to biosecurity compliance in parts of northeastern Nigeria [11], but adequate biosecurity measures were reported in commercial poultry farms in the same region while some backyard farms lacked such measures [9]. In Egypt, biosecurity measures were rarely implemented in small-scale commercial production units [29]. In Jos, most small-scale farms returned positive responses (>60%) on several indicators of important biosecurity events. Deficiency in biosecurity compliance was remarkable when positive responses dropped to <60% in aspects of providing parking lot outside the farm and functional footbath at farm boundaries. Furthermore, vehicles drove into farm premises without washing and disinfection; farm workers had residences outside the farm premises in >50% of farms and some farms used second-hand equipment like egg crates. It was necessary to avoid these areas of biosecurity failures [4]. The risks associated with avian influenza outbreaks in Nigeria were receiving visitors to the farm, purchasing of live poultry or products, and workers living outside the farm premises [10, 30, 31]. Therefore, biosecurity compliance is a compact practice which should not give room for any gaps in the production system for entry or exit of infectious agents [7].

The biosecurity scoring system adopted in this report incorporated a broad scope of indicators of a variety of biosecurity events, thereby making it comprehensive for the local production system. Our biosecurity scoring system scored each indicator equally in a positive or negative sense [21] and offered an average BS for a farm but differed from other scoring systems with scores of 0–3 [22] or 1–3 [8, 20] for an indicator. The current scoring system provided the quantitative means to determine the extent production system and flock size influenced the biosecurity scores of farms. Semicommercial farms had significantly higher BS than strictly commercial farms; and it is presumed that employed labour in strictly commercial farms may be nonchalant about aspects of biosecurity in the absence of owners' supervision and appropriate motivation. On-farm biosecurity was reported to be affected by the level of motivation derived from the information on costs and accrued benefits [14]. The flock size did not significantly influence BS, but highest mean BS was in farms with the largest flock size. The contribution to the production capacity of the poultry sector was greater with larger compared to smaller farms; and as a result, enhanced efficiency of the production system was expected as regards investments in biosecurity measures to mitigate production losses. Dorea et al. [28] reported that flock size did not affect the standard of biosecurity protocols implemented, maybe because the awareness of biosecurity was broad and cut across the spectrum of small and large scale farm managements as the case was in this study. However, other reports indicated that farmers with larger farm areas and flock sizes tended to have enhanced biosecurity practices [8, 13].

Occurrence of outbreaks of communicable disease on farms decreased with increasing BS, thereby, affirming the importance of biosecurity in the production system's disease control. There have been reports of reduction of infectious disease outbreaks with standard biosecurity protocols [6, 32]. Biosecurity failures seemed to be a greater challenge in deep litter than battery cage farms, since outbreaks were significantly associated with deep litter system. The need for disease control in deep litter is amplified by the fact that farmers rarely use battery cage because of cost. Disease transmission occurs on deep litter by contact through inhalation of aerosolized particles and ingestion of contaminated feed and water [33–36]. In order to prevent infections, farmers (74% of respondents) engage in prophylactic treatments of apparently healthy birds, sometimes without proper veterinary supervision and with adverse consequences [37, 38]. Development of antimicrobial resistance may occur as a result of this practice [38] and antimicrobial residues have been reported in poultry meat and eggs when drug withdrawal period is not observed with the implication of food chain contaminations [39, 40].

All the farms were registered for veterinary services and most of them sought for such services only in the event of health care challenges. Regular veterinary farm visits through retainership programs was not often in practice. Farmers call for veterinary investigation of mortality of chickens and necropsies are usually carried out as part of the diagnostic process. Ideally, the carcasses are moved in a biosecure manner to veterinary diagnostic facilities. Some farms, in this study, reported carrying out necropsies on the farm and this was considered a negative biosecurity event especially where disinfection and appropriate disposal of carcasses were not ascertained. Most farmers in Jos sold dead chickens which were subsequently dressed and cooked for consumption by dogs. This practice is considered to have biosecurity implications and was presumed to be an effort to reduce financial loss. By scouting for dog owners who have established demand windows, farmers enhance the value chain in the industry. Carcasses of dead chicken on poultry farms were reported to be disposed by incineration, deep burial, or dumping as refuse [9]. Dumping of carcasses was rare and farmers knew the associated biosecurity risks of such act. Few farmers had on-farm carnivorous pets (dogs and cats) and were not aware of negative biosecurity implications of the pets having possible chance of eating unprocessed dead carcasses.

In conclusion, the questionnaire-based study revealed that the intensive chicken production system in Jos lacked integration of all inputs and consisted mostly of small farms, but few larger farms had aggregate bird population exceeding those of small farms. Semicommercial farms had higher BS than strictly commercial farms. Outbreaks of communicable disease negatively correlated with BS in deep litter system, and farmers engaged in prophylactic treatments to reduce production loses that might arise from biosecurity failures. The weak points in the biosecurity were identified and could be strengthened by providing the farmers information and focus group training to fill the gap in understanding of the scope of engagement of biosecurity practice as an important phenomenon in preventive poultry health.

## Figures and Tables

**(a):** 

	Number (%) of farms	Number (%) of birds
Reason for keeping birds		
Commercial	47 (58.8)	71843 (75.3)
Family use (noncommercial)	3 (3.7)	150 (0.2)
Both (semicommercial)	30 (37.5)	23400 (24.5)
Total	80 (100.0)	95393 (100.0)
Poultry type		
Broiler	11 (13.8)	3701 (3.9)
Layer	51 (63.8)	46540 (48.8)
Both broiler and layer	17 (21.2)	44672 (46.8)
Cockerel and layer	1 (1.2)	480 (0.5)
Total	80 (100.0)	95393 (100.0)
Number of birds per farm		
<200	9 (11.2)	743 (0.8)
200–500	29 (36.2)	10460 (11.0)
501–1000	20 (25.0)	15795 (16.5)
1001–2000	15 (18.8)	24145 (25.3)
2000–10000	5 (6.3)	14950 (15.7)
>10000	2 (2.5)	29300 (30.7)
Total	80 (100.0)	95393 (100.0)
Age of birds at study time		
<13 wks	23 (28.8)	26966 (28.3)
13–17 wks	3 (3.7)	1900 (2.0)
≥18 wks	54 (67.5)	66527 (69.7)
Total	80 (100.0)	95393 (100.0)

**(b):** 

	Number (%) of farms	Number (%) of birds
Housing system		
Deep litter	69 (86.3)	73545 (77.1)
Battery cage	10 (12.5)	7548 (7.9)
Both	1 (1.2)	14300 (15.0)
Total	80 (100.0)	95393 (100.0)
Source of day-old chicks		
Obasanjo	16 (20.0)	30616 (32.1)
Zartech	28 (35.0)	37302 (39.1)
Zartech and Agrited	2 (2.5)	4500 (4.7)
Agrited	5 (6.3)	3350 (3.5)
ECWA	6 (7.5)	5398 (5.7)
Amo	6 (7.5)	4280 (4.5)
Chi	7 (8.7)	4600 (4.8)
Unknown	10 (12.5)	5347 (5.6)
Total	80 (100.0)	95393 (100.0)
Sources of feed		
Commercial	68 (85.0)	82896 (86.9)
Vital	18 (26.5)	12370 (14.9)
Livestock	19 (29.4)	34307 (41.4)
Amo	6 (8.8)	4242 (5.1)
Top	2 (2.9)	680 (0.8)
Hybrid	16 (23.5)	10622 (12.8)
Vital and top	1 (1.5)	15000 (18.1)
Unknown	5 (7.4)	5675 (6.9)
On-farm (local) preparation	6 (7.5)	6850 (7.1)
Both	6 (7.5)	5647 (6.0)
Total	80 (100.0)	95393 (100.0)

**Table 2:** Frequency of biosecurity events on poultry farms in Jos, Nigeria.

Indicators of biosecurity events	Number (%) of responses	Number (%) of farms with “yes” response
Events outside the premises		
Awareness of biosecurity practices	79 (98.8)	67 (84.8)
Density of (>5) farmers in neighbourhood	79 (98.8)	21 (26.6)
Water bodies for migratory birds in neighbourhood	79 (98.8)	16 (20.2)
Certified sources of quality chicks	76 (95.0)	52 (68.4)
Parking lot outside the farm premises	77 (96.3)	42 (54.5)
Acquisition of second-hand equipment	79 (98.8)	5 (6.3)
Farm boundary events		
Fencing with gates	78 (97.5)	66 (84.6)
Washing/disinfection of vehicles	76 (95.0)	17 (22.3)
Functional^†^ footbath at entry point	78 (97.5)	44 (56.4)
Visitors allowed into premises	78 (97.5)	37 (47.4)
Events between farm boundary and poultry house		
Presence of good feed storage facility	79 (98.8)	75 (94.9)
Appropriate carcass disposal	80 (100.0)	78 (97.5)
On-farm necropsy	79 (98.8)	25 (31.6)
Certified commercial feed sources only	80 (100.0)	68 (85.0)
On-farm carnivores (dogs and cats)	79 (98.8)	7 (8.9)
Hand washing/shower before and after handling birds	79 (98.8)	65 (82.3)
Rodent-proof	78 (97.5)	74 (94.9)
Residence of farm workers within premises	79 (98.8)	39 (49.4)
Functional^†^ footbath at entrance of poultry house	77 (96.3)	56 (72.7)
Events inside poultry house		
Separation of poultry types	80 (100.0)	78 (97.5)
Separation of birds according to age	80 (100.0)	79 (98.8)
Proper ventilation	80 (100.0)	79 (98.8)
Availability of clean water	77 (96.3)	76 (98.7)
Appropriate bedding material	69 (86.3)	66 (95.7)
Dry bedding	65 (81.3)	64 (98.5)
Frequent changing of bedding	66 (82.5)	59 (89.4)
Birds occasionally allowed to move out of the poultry house	78 (97.5)	3 (3.8)
Washing/disinfecting poultry house prior to restocking	78 (97.5)	76 (97.4)
Practice of all-in all-out management system	77 (96.3)	69 (89.6)
Washing feeders/drinkers regularly	78 (97.5)	77 (98.7)
Disinfecting feeders/drinkers regularly	75 (93.8)	48 (64.0)
Isolation of apparently sick birds	76 (95.0)	74 (97.4)
Prophylactic chemotherapy to apparently healthy birds	77 (96.3)	57 (74.0)
Consultation of veterinarians only in the event of problems	78 (97.5)	60 (76.9)

^†^Functional footbath comprises (a) presence of the footbath and (b) frequent (daily or once every two days) replenishment of the same.

**Table 3:** Effects of reasons for keeping poultry and number of birds per farm on biosecurity scores.

	Biosecurity score^*∗*^ (number of farms^‡^)
Reason for keeping poultry	
Commercial	0.75 ± 0.10^a^ (32)
Family use only (noncommercial)	0.82 ± 0.07^ab^ (3)
Both (semicommercial)	0.87 ± 0.05^b^ (28)
Number of birds per farm	
<200	0.80 ± 0.07^a^ (7)
200–500	0.81 ± 0.11^a^ (23)
501–1000	0.79 ± 0.13^a^ (15)
1001–2000	0.80 ± 0.10^a^ (12)
2001–10000	0.85 ± 0.03^a^ (4)
>10000	0.88 ± 0.02^a^ (2)

^a,b^Unmatched superscripts on means ± standard deviations indicate significant (*p* < 0.05) difference in the column for each set of variables.

^*∗*^Average score on correct responses on biosecurity indicators on a farm, where incorrect and correct scores are 0 and 1, respectively.

^‡^Farms with ≥ 34 responses on biosecurity (*n* = 63).

**Table 4:** Number (%) of farms with various scores on biosecurity in relation to occurrence of disease outbreak^#^.

Biosecurity score^*∗*^	Number (%) of farms	
	All responding farms^‡^	Farms with disease outbreak
<0.5	0	0
0.50–0.70	11 (17.5)	9 (81.8)
0.71–0.90	45 (71.4)	24 (53.3)
0.91–1.00	7 (11.1)	3 (42.9)

<0.5–1.00	63 (100)	36 (57.1)

^*∗*^Average score on correct responses on biosecurity indicators on a farm, where incorrect and correct scores are 0 and 1, respectively.

^‡^Farms with ≥ 34 responses on biosecurity.

^#^Inverse relationship between biosecurity scores in first column with percentages of disease outbreak in third column is computed to give a correlation coefficient of −0.97.

**Table 5:** Effect of housing system on the occurrence of disease outbreaks on farms.

Housing system	Number (%) of farms	
	All responding farms	Farms with disease outbreak
Deep litter	69 (86.3)	38 (55.1)^a^
Battery cage	10 (12.5)	0 (0)^b^
Both (deep litter and battery cage)	1 (1.2)	1 (100.0)^ab^

Total	80 (100.0)	39 (48.8)

^a,b^Unmatched superscripts indicate significant (*p* < 0.05) difference in the column.
